# Platelet Phenotype and Function in the Setting of Pediatric Extracorporeal Membrane Oxygenation (ECMO): A Systematic Review

**DOI:** 10.3389/fcvm.2019.00137

**Published:** 2019-09-18

**Authors:** Hui Ping Yaw, Suelyn Van Den Helm, Graeme MacLaren, Matthew Linden, Paul Monagle, Vera Ignjatovic

**Affiliations:** ^1^Department of Haematology Research, Murdoch Children's Research Institute, Melbourne, VIC, Australia; ^2^Department of Paediatrics, The University of Melbourne, Melbourne, VIC, Australia; ^3^Paediatric Intensive Care Unit, The Royal Children's Hospital, Melbourne, VIC, Australia; ^4^Cardiothoracic Intensive Care Unit, National University Health System, Singapore, Singapore; ^5^School of Biomedical Sciences, The University of Western Australia, Perth, WA, Australia; ^6^Department of Clinical Haematology, The Royal Children's Hospital, Melbourne, VIC, Australia

**Keywords:** platelet, extracorporeal membrane oxygenation (ECMO), pediatric, bleeding, clotting, phenotype and function

## Abstract

**Background:** Despite increasing technical improvement and extracorporeal membrane oxygenation (ECMO)-related knowledge over the past three decades, morbidity and mortality associated with bleeding and clotting complications remain high in pediatric patients undergoing ECMO. Platelets, a key element of the coagulation system, have been proposed to be the main cause of coagulopathy in the setting of ECMO. This systematic review aims to summarize and discuss the existing knowledge of platelet phenotype and function in the pediatric ECMO population.

**Methods:** A systematic review was conducted for the Embase, Medline, and PubMed databases following the Preferred Reporting Items for Systematic Reviews and Meta-Analysis (PRISMA) guidelines.

**Results:** The detailed study selection process yielded a total of 765 studies and only 3 studies that fulfilled the selection criteria were included in this review. Techniques used to assess platelet function in the three existing studies included platelet aggregometry, flow cytometry, and thromboelastography-platelet mapping. The finding that is common to the three studies is reduced platelet function in pediatric patients during ECMO either compared to before the initiation of ECMO or in non-survivors compared to survivors. Two studies demonstrated reduced platelet aggregation that are irreversible by platelet transfusion during ECMO. Two studies reported bleeding events and mortality in children on ECMO and none of the studies investigated thrombotic events.

**Conclusions:** This systematic review demonstrates the extremely limited information available for platelet phenotype and function in the pediatric ECMO population. Evidence from the existing literature suggests reduced platelet aggregation and increased platelet activation in children during ECMO. However, this needs to be interpreted with care due to the limitations associated with the techniques used for platelet function testing. Furthermore, the association between platelet dysfunction and clinical outcomes in the pediatric ECMO population remains elusive. Multiple research gaps have been identified when it comes to the knowledge of platelet phenotype and function of children on ECMO, highlighting the need for robust, well-designed studies in this setting.

## Introduction

Extracorporeal membrane oxygenation (ECMO) is a modified form of cardiopulmonary bypass that aims to provide short to medium-length cardiac and/or respiratory support to patients. Despite the improvement in technologies and clinical practice over the years, bleeding, and/or thrombosis complications remain a major cause of morbidity and mortality in children on ECMO, with an overall survival rate of 54.9% (1). Using definitions of bleeding and thrombosis from the Extracorporeal Life Support Organization (ELSO), a study by Dalton et al. on 514 patients showed that bleeding is common in children on ECMO, with a rate of 70.2%, including intracranial hemorrhage in 16% of cases (1). Thrombotic events occurred in 37.5% of the patients, with 31.1% of patients requiring circuit component changes due to thrombosis (1).

The artificial surface and shear stress originating from the ECMO system have been proposed to be the main cause of coagulopathy seen in ECMO patients, via mechanisms such as activation of the hemostatic system. Modification of platelet function, a key element of the clotting system, has been suggested to be the main determinant of ECMO-induced bleeding and/or thrombosis in both the adult and pediatric ECMO population. Particularly, shear stress-induced platelet activation and loss of important platelet receptors have been reported in multiple studies for adults on ECMO and the resultant platelet dysfunction has been associated with increased thrombotic propensity and reduced hemostatic capacity (2–5).

Differences in platelet function and proteome between healthy adults and healthy children have been evaluated in multiple studies (6–8). Existing research in the ECMO population focuses on adults. However, the results may not be applicable to children due to the physiological differences between adults and children and the complex nature of ECMO. In addition, differences in response toward unfractionated heparin (UFH) (the main anticoagulant used in the setting of ECMO) between adults and children have also been documented (9, 10) and may be associated with the increased risk of anticoagulation-related complications in the pediatric ECMO cohort. Furthermore, differences in the clinical outcome between the adult and children ECMO patients (11) may also indicate differences in the pathophysiological development of coagulopathy between these two patient cohorts. Currently, there is very limited knowledge specific to platelet phenotype and function in children on ECMO (12–14) and there is no systematic review that summarizes the existing evidence for this population. On the other hand, existing studies relevant to platelet function and acquired von Willebrand syndrome were discussed previously for the adult ECMO population (15, 16). Hence, this systematic review aims to assemble and elaborate on existing studies focusing on platelet phenotype and function in children on ECMO.

## Methods

### Search Strategy

This systematic review was conducted based on the Preferred Reporting Items for Systematic Reviews and Meta-Analyses (PRISMA) guidelines (17). The Embase, Medline and PubMed databases were searched systematically on August 1, 2019 to identify studies in human and limited to the English language only. The following search terms were used for each of the database:

**Embase:** (^*^extracorporeal oxygenation OR ^*^membrane oxygenator OR extracorporeal-membrane-oxygenation OR extra-corporeal-membrane-oxygenation OR extracorporeal-life-support OR extra-corporeal-life-support OR extracorporeal-cardiopulmonary-resuscitation OR extra-corporeal-cardiopulmonary-resuscitation OR ecmo OR ecls OR ecpr) AND (thrombocyte OR thrombocyte activation OR antithrombocytic agent OR platelet count OR blood clotting parameters OR thrombocytopheresis OR thrombocytopenia OR thrombocytosis OR thrombocyte transfusion OR fluoroimmunoassay OR ^*^flow cytometry OR flow-cytometry OR aggregometry or platelet^*^ OR thrombocyt^*^ OR ^*^thrombocyte function OR ^*^thrombocyte dysfunction OR thrombocyte-count) AND (newborn^*^ OR baby OR babies OR neonat^*^ OR infan^*^ OR toddler^*^ OR pre-schooler^*^ OR preschooler^*^ OR kindergarten OR boy OR boys OR girl OR girls OR child OR children OR childhood OR adolescen^*^ OR pediatric^*^ OR paediatric^*^ OR youth^*^ OR teen OR teens OR teenage^*^ OR school-aged^*^ OR school-child^*^ OR school-girl^*^ OR school-boy^*^ OR schoolgirl^*^ OR schoolboy^*^).
**Medline:** (extracorporeal membrane oxygenation OR extracorporeal circulation OR oxygenators, membrane) AND (blood platelets OR platelet activation OR platelet aggregation inhibitors OR platelet count OR platelet function tests OR plateletpheresis OR thrombocytopenia OR thrombocytopenia OR thrombocytosis OR platelet transfusion OR fluoroimmunonoassay OR flow cytometry OR flow-cytometry or aggregometry or platelet^*^ or thrombocyt^*^) AND (newborn^*^ OR baby OR babies OR neonat^*^ OR infan^*^ OR toddler^*^ OR pre-schooler^*^ OR preschooler^*^ OR kindergarten OR boy OR boys OR girl OR girls OR child OR children OR childhood OR adolescen^*^ OR pediatric^*^ OR paediatric^*^ OR youth^*^ OR teen OR teens OR teenage^*^ OR school-aged^*^ OR school-child^*^ OR school-girl^*^ OR school-boy^*^ OR schoolgirl^*^ OR schoolboy^*^).
**PubMed**: #1 (Extracorporeal-oxygenation OR Extra-corporeal-oxygenation OR Extracorporeal-membrane-oxygenation OR Extra-corporeal-membrane-oxygenation OR extracorporeal-life-support OR extra-corporeal-life-support OR extracorporeal-cardiopulmonary-resuscitation OR extra-corporeal-cardiopulmonary-resuscitation OR ECMO OR ECLS OR ECPR OR Extracorporeal-circulation OR Extra-corporeal-circulation OR membrane-oxygenator^*^) AND (Platelet^*^ OR thrombocyt^*^ OR Platelet-count OR Platelet-activation^*^ OR thrombocyte-activation^*^ OR Platelet-function^*^ OR blood-clotting-parameter^*^ OR Thrombocytopaenia^*^ OR Thrombocytopenia^*^ OR Thrombo-cytopaenia^*^ OR Thrombo-cytopenia^*^ OR Platelet-transfusion^*^ OR thrombocyte-transfusion^*^ OR Flow-cytometry OR fluoroimmunoassay^*^ OR fluoro -immunoassay^*^ OR Platelet-aggregation OR antithrombocytic-agent^*^ OR anti-thrombocytic-agent^*^ OR Plateletpheresis OR Thrombopheresis OR thrombocytopheresis OR aggregometry) AND (NOTNLM OR publisher[sb] OR inprocess[sb] OR pubmednotmedline[sb] OR indatareview[sb] OR pubstatusaheadofprint)) NOT ((animal OR animals OR rat OR rats OR mouse OR mice OR rodent^*^ OR murine OR sheep) NOT (human OR humans OR newborn^*^ OR baby OR babies OR neonat^*^ OR infan^*^ OR toddler^*^ OR pre-schooler^*^ OR preschooler^*^ OR kindergarten OR boy OR boys OR girl OR girls OR child OR children OR childhood OR adolescen^*^ OR pediatric^*^ OR paediatric^*^ OR youth^*^ OR teen OR teens OR teenage^*^ OR school-aged^*^ OR school-child^*^ OR school-girl^*^ OR school-boy^*^ OR schoolgirl^*^ OR schoolboy^*^).
#2 (Case Reports[ptyp]) OR (Review[ptyp]).
#3 #1 NOT #2.

### Study Selection

Inclusion criteria: (I) pediatric patients (0–18 years), (II) primary research, (III) platelet function assessed, (IV) English language, and (V) human study.

Exclusion criteria: (I) adult patients (>18 years), (II) review, case report, guideline, editorial correspondence, or conference abstract, (III) only platelet count measured, and (IV) animal study.

## Results

**Figure 1:**
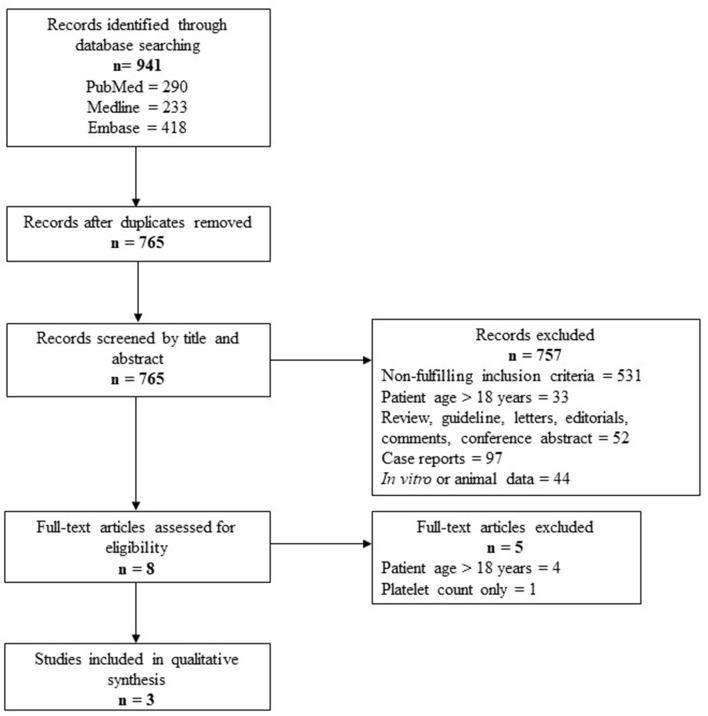
Summary of the study selection process for the systematic review.

A total of 765 studies were identified with this systematic review search strategy. The majority of the studies (*n* = 757) that did not fulfill the inclusion criteria were excluded, including case reports that did not focus on assessing platelet phenotype and function. Eight studies were included for full-text screening and five of them were excluded for either patients aged >18 years or had assessed only platelet count in the study. A total of three studies matched the inclusion criteria and were summarized in this review. The steps used following the PRISMA guidelines are outlined in Figure 1. The summary for each of the included studies is included in Table 1.

**Table 1:** The summary of the studies included in the systematic review for platelet phenotype and function in pediatric patients on ECMO.

**References**	**Sample size**	**Age**	**Study design**	**Mode of ECMO**	**Indication**	**Duration of ECMO**	**Time points**	**Clinical events (number of events)**	**Therapeutic agents with reported anti-platelet effects**	**Monitoring of platelet function (method)**	**Results**	
											**Platelet count**	**Platelet function**
Robinson et al. (12)	*n* = 10	Reported: Mean (range) = 3 (1–8) days Calculated: Range = 7 days	Prospective	Not reported	Respiratory failure (*n* = 10)	Reported: Range = 4–16 days Calculated: Range = 12 days	Baseline 15 min 60 min after initiation of ECMO 15 min 60 min after platelet transfusion	**Bleeding** *n* = 0 **Thrombosis** Not reported **Mortality** Not reported	Not reported	**Flow cytometry** (Surface-bound GPIb and IIIa) **Platelet aggregation** (Whole blood aggregometer using collagen, ADP and ristocetin as agonists) **Bioluminescent assay** (Luciferase detection of platelet ATP release in response to collagen and thrombin)	Significant reduction 60 min after ECMO initiation compared to baseline Decreased to pre-transfusion value by 60 min after transfusion	Significant reduction 15 min after ECMO initiation compared to baseline No change in GPIb and IIIa expression Platelet aggregation was not improved by platelet transfusion but resolved within 8 h after decannulation from ECMO
Cheung et al. (13)	*n* = 10	Reported: Mean ± SEM = 25 ± 4 h postnatal age Calculated: Range = 48 h of postnatal age	Prospective	Not reported	Meconium aspiration syndrome (*n* = 5) Congenital diaphragmatic hernia (*n* = 5)	Reported: Mean ± SEM = 91 ± 4 h Calculated: Range = 157 h	Baseline 1 h 2 h 4 h 12 h 24 h after initiation of ECMO 1 h before decannulation 24 h post-decannulation	**Bleeding** *n* = 1 (10%) **Thrombosis** Not reported **In-hospital mortality** *n* = 2 (20%)	Not reported	**Platelet aggregation** (Whole blood aggregometer using collagen as agonist) **ELISA** (Soluble P-selectin) **Zymography** (Gelatinolytic activities of plasma matrix metalloproteinases, MMP-2, and MMP-9)	Significant reduction 1 h after ECMO initiation and 24 h after decannulation; no significant difference 4, 12, and 24 h after ECMO initiation and 1 h before decannulation compared to baseline	Significant reduction 1, 2, 4, 12, and 24 h after ECMO initiation; no significant difference 1 h before and 24 h after decannulationcompared to baseline Significant increase in soluble P-selectin 4, 12, and 24 h after ECMO initiation; no significant difference 1 and 2 h after ECMO initiation and 24 h after decannulation compared to baseline
Saini et al. (14)	*n* = 24	Reported: Median (IQR) = 9 (1–70) months Calculated: N/A	Retrospective	VA-ECMO *n* = 18 (75%) VV-ECMO *n* = 6 (25%)	Acute respiratory distress syndrome (*n* = 4) Pulmonary hypertension (*n* = 4) Congenital diaphragmatic hernia (*n* = 2) Myocarditis/dilated cardiomyopathy (*n* = 3) Post-operative (*n* = 11)	Reported: Median (IQR) = 8 (6–10) days Calculated: N/A	24 h prior to a severe bleeding event Prior to ECMO decannulation	**Bleeding** *n* = 10 (42%) severe **Thrombosis** Not reported **In-hospital mortality** *n* = 13 (54%)	Inhaled nitric oxide, milrinone, and H_2_ blockers	**Thromboelastography** (Platelet-mapping using ADP and AA)	No significant difference between patients who had severe bleeding and those who had not Significant reduction in non-survivors compared to survivors	Significant reduction in patients who had severe bleeding compared to those who had not Significant reduction in non-survivors compared to survivors

*AA, arachidonic acid; ADP, adenosine diphosphate; ATP, adenosine triphosphate; ECMO, extracorporeal membrane oxygenation; GPIb, glycoprotein Ib; ELISA, enzyme-linked immunosorbent assay; GPIIIa, glycoprotein IIIa; IQR, inter-quartile range; N/A, not available; PLC, platelet count; SEM, standard error of the mean; VA-ECMO, venoarterial ECMO; VV-venovenous ECMO*.

## Discussion

This systematic review demonstrates the absolute lack of studies that have focused on platelet phenotype and function in pediatric patients on ECMO. Such a lack of information in this population could reflect challenges in ethical approval and limited availability of blood sample volume for pediatric research. The main techniques used in the three existing studies included aggregometry, flow cytometry, and thromboelastography-platelet mapping. The common findings are reduced platelet function during ECMO. Particularly, evidence showed that the acquired platelet dysfunction during ECMO is not resolved by platelet transfusion. The relationship of plasma problems such as acquired von Willebrand's disease to the platelet dysfunction is unknown. In addition, the association between platelet dysfunction and bleeding and/or thrombosis commonly seen in children on ECMO has not been investigated to date.

Two out of the three studies utilized platelet aggregometry to investigate platelet function using agonists such as collagen, ADP and ristocetin (12, 13). Both studies reported reduced platelet aggregation in pediatric patients during ECMO despite the platelet transfusions given to the patients during ECMO. Robinson et al. reported reduced platelet aggregation in response to ADP and ristocetin but not for collagen within 15 min upon initiation of ECMO (12) whereas Cheung et al. have shown a decrease in platelet aggregation in response to collagen in a time-dependent manner starting from within 24 h upon cannulation for ECMO (13). The difference in collagen-induced platelet aggregation between the two studies may be related to the difference in concentration of collagen (5 vs. 10 μg/mL) used.

Despite being the gold standard in platelet function testing for many years, the applicability of aggregometry to the pediatric patients is often limited by the requirement of a large volume of blood. Specifically, platelet aggregometry usually requires 0.5–2 mL of blood which is a significant volume of blood to be withdrawn on multiple occasions from children who are critically ill and are sampled very frequently for a variety of clinical assays. Dalton et al. showed in children on ECMO that laboratory blood sampling is the primary cause of significant blood loss and is the main reason of transfusion in 42.2% of the studied population (1). Furthermore, multiple studies have suggested a strong association between platelet aggregometry and platelet count (18–20), which is problematic given that thrombocytopaenia is commonly found in children on ECMO. A study by Fernia et al. showed that light transmission aggregometry can be affected by platelet counts of <150 × 10^9^ /L (20). None of the existing studies have reported whether any cut-off value was set for the platelet count for patients to be included in the analysis of platelet function using platelet aggregometry. Hence, results from the existing studies need to be interpreted with care.

In recent years, whole blood flow cytometry analysis of platelet function has gained attention in the pediatric setting for the requirement of only a minimal amount of blood and enabling analysis of platelets in their physiological environment, in the presence of the other blood cells with minimal manipulation. However, multiple technical challenges such as the requirement for trained-personnel and expensive reagents have limited its usage in a wider population. Only one study utilized flow cytometry to evaluate the expression of GPIb and IIIa, platelet markers important for adhesion and aggregation (12). There was no difference in the expression of these two receptors during ECMO compared to before initiation of ECMO. However, this study is now over 25 years old and flow cytometry techniques have improved considerably. In addition, these results must be interpreted with care as platelets are highly subjected to pre-analytical activation and have a limited processing window. Optimization of the flow cytometry methods for evaluation of platelet phenotype and function is important and this was not indicated as part of the study by Robinson et al. (12). Flow cytometric analysis of surface receptors on circulating platelets in adults on mechanical circulatory support (MCS) showed that ECMO patients had significantly lower expression of GPIbα and GPVI (platelet adhesion receptors) compared to healthy individuals (21). Whether the same changes occur in children of different ages remains to be determined.

In addition to platelet aggregometry and flow cytometry, thromboelastography-platelet mapping (TEG®-PM) has gained popularity as a platelet function assay in recent years and few centers have incorporated this system for hemostasis monitoring in children on ECMO (22). By using TEG®-PM, Saini et al. reported severe platelet dysfunction in more than 75% of the patient cohort (14). Using Multiplate® analyser with ADP and ristocetin, Nair et al. demonstrated 50–72% incidence of platelet dysfunction in the adult ECMO population (23). Furthermore, platelet count and TEG®-PM were shown to be a better predictor of severe bleeding and mortality compared to the activated clotting time in children on ECMO (14). A study by Weitzel et al. on the effects of cardiopulmonary bypass on cardiac surgery patients [mean (standard deviation) age: 58 (17)] also found reduced platelet aggregation in response to collagen, ADP and AA in the patient cohort and reported 83% sensitivity and 68% specificity for TEG®-PM to predict post-operative bleeding (24).

Thrombocytopaenia is the main trigger of platelet administration in ECMO patients and has been shown to be an important predictor of hemorrhage (25–27). Nevertheless, a study by Reed and Rutledge found no correlation between platelet level and hemorrhage or thrombosis in a cohort of deceased pediatric ECMO patients (28). On average, children on ECMO received 1.3 platelet transfusions per day to maintain platelet level of >100 × 10^9^/L (29). However, frequent platelet transfusions in ECMO patients with excessive bleeding who had normal platelet counts may indicate occurrence of platelet dysfunction. Hence, in addition to the platelet count, it will also be important to monitor platelet function, which has been proposed to be the main cause of coagulopathy in ECMO patients.

In recent years, interactions between platelets and leukocytes have gained increased attention for their important roles in the cross-talk between the hemostatic and inflammatory systems. Currently, there are no studies that have investigated platelet-leukocyte interactions in children or adults on ECMO. Initiation of ECMO is known to induce inflammation via different mechanisms (30, 31) and elevated monocyte-platelet levels have been found in various thromboinflammatory conditions such as cardiovascular diseases (32, 33). Hence, future research focusing on investigating platelet-leukocyte interactions will be important for better understanding of how the interaction between the hemostatic and inflammatory systems may be associated with the development of clinical events in the pediatric ECMO population.

## Conclusions

This systematic review revealed the existing research gaps in platelet function for children on ECMO. Existing studies are limited by small sample sizes and the types of platelet function tests performed. Pediatric ECMO patients represent a complex patient cohort with diverse etiologies and complications, resulting in pathological bleeding, and clotting. Hence, it will be crucial for future assessment of platelet function to investigate platelet phenotype and function from multiple aspects tailored to the extensive roles of platelets that are not only limited to coagulation but also as immune and inflammatory cells. In particular, future research using the multi-advantageous whole blood flow cytometry should focus on optimized and robust experimental design to ensure standardization and validity of the method used. Furthermore, it will be important to take into account the multiple patient-related factors such as interventions and procedures leading up to commencement of ECMO, age, and duration on ECMO for analysis in relation to the complex nature of this patient cohort.

## Author Contributions

All authors listed have made a substantial, direct and intellectual contribution to the work, and approved it for publication.

### Conflict of Interest Statement

The authors declare that the research was conducted in the absence of any commercial or financial relationships that could be construed as a potential conflict of interest.
